# Mesenchymal Stem Cell Therapy for Neurological Complications of Prematurity: A Narrative Review

**DOI:** 10.3390/ph19030464

**Published:** 2026-03-12

**Authors:** Hua (Hannah) Yep, Jennifer H. Bae, George A. Wen, Sangel Gomez, Alexandra Tsivitis, Robert P. Moore, Helen Hsieh, Sergio D. Bergese

**Affiliations:** 1 Department of Anesthesiology, Stony Brook University Hospital, Stony Brook, NY 11794, USA; hua.yep@stonybrookmedicine.edu (H.Y.); sangel.gomez@stonybrookmedicine.edu (S.G.); alexandra.tsivitis@stonybrookmedicine.edu (A.T.); robert.moore5@stonybrookmedicine.edu (R.P.M.); 2 Renaissance School of Medicine, Stony Brook University, Stony Brook, NY 11794, USA; jennifer.bae@stonybrookmedicine.edu (J.H.B.); george.wen@stonybrookmedicine.edu (G.A.W.); 3 Department of Surgery, Stony Brook University Hospital, Stony Brook, NY 11794, USA; helen.hsieh@stonybrookmedicine.edu

**Keywords:** mesenchymal stem cells, encephalopathy of prematurity, stem cell therapy, extracellular vesicles, hit and run, neonate, pre-term

## Abstract

**Background**: Preterm birth is a leading cause of neonatal mortality and long-term disability worldwide. Injury in premature infants is demonstrated by disrupted organ development from inflammation, oxidative stress, hypoxia, and impaired vascular maturation. Current therapies largely provide supportive care and do not directly promote tissue regeneration. Mesenchymal stem cell (MSC)-based therapies have emerged as a potential strategy to enhance endogenous repair across organ systems commonly affected by prematurity. **Results**: Evidence indicates that MSCs exert therapeutic effects primarily through transient paracrine signaling rather than long-term engraftment. Following administration, MSCs release cytokines, growth factors, and extracellular vesicles that reduce inflammation, promote angiogenesis, and support tissue repair. In preclinical models of neonatal brain injury, MSC therapy has been associated with improved oligodendrocyte maturation and reduced white matter injury. Early clinical trials in neonatal encephalopathy demonstrate feasibility and short-term safety of both autologous and allogeneic cell products. However, studies remain limited by small sample sizes and short follow-up. Cell-free approaches using MSC-derived extracellular vesicles may offer similar biological benefits with potentially lower safety and regulatory concerns. **Conclusions**: MSC-based therapies represent a promising regenerative approach for complications of prematurity. Rigorous, large-scale trials with standardized protocols and long-term follow-up are necessary to clarify efficacy, optimize delivery strategies, and define safety in this vulnerable population.

## 1. Introduction

Preterm birth affects approximately 1 in 10 live births worldwide and has a profound impact on patients, families, and society. It is a major source of pediatric morbidity and mortality. Prematurity is one of the leading causes of mortality in children under five years of age [1].

Despite advances in medical care, complications associated with prematurity accounted for nearly 1 million neonatal deaths in 2022 [1]. Earlier preterm births are associated with more severe complications. Survivors of prematurity often experience multiorgan issues including neurodevelopmental impairment, bronchopulmonary dysplasia (BPD), and necrotizing enterocolitis (NEC). In a population-based matched cohort study, individuals born preterm faced an elevated risk of mortality extending into their third and fourth decades of life [2]. Limiting these complications could have a profound impact on the health and quality of life of millions of children. Current therapies for prematurity focus on prevention and life-sustaining treatments in the setting of multisystem organ immaturity [3].

Stem cell therapy shows promise as a novel approach for treating several prematurity-related sequelae; there are encouraging preclinical results supporting its potential for clinical use [4]. Stem cells are undifferentiated cells that self-renew and/or differentiate into specialized cell types. Disruption during key developmental stages in premature patients results in underdevelopment of organs; therefore, stem cell therapy may promote regeneration and repair in these immature tissues. However, there are significant barriers to unleashing stem cell therapy in this vulnerable cohort including the need for more safety data, clinical impact, and an understanding of the impact of regulatory concerns.

This review discusses the pathophysiology of prematurity, the principles of stem cell therapies, and evaluates the therapeutic potential of stem cell interventions across multiple organ systems in premature infants, including long-term outcomes, future directions, and ethical considerations.

## 2. Principles of Stem Cells

Stem cell research is at the forefront of modern developmental biology and regenerative medicine due to the unique ability of stem cells to self-renew and differentiate into specialized cell types [5]. Defined by their capacity for indefinite proliferation and multilineage differentiation, stem cells play essential roles in tissue maintenance, repair, and embryonic development [6]. Over the past several decades, advances in stem cell biology—spanning embryonic, adult, and induced pluripotent stem cells—have significantly deepened our understanding of cellular plasticity. These discoveries have driven the development of novel therapeutic strategies for a wide range of diseases and injuries, with the goal of promoting endogenous and exogenous tissue repair [7]. As research progresses, the technical and ethical challenges surrounding stem cell science remain central to its continued evolution. 

**Figure 1:**
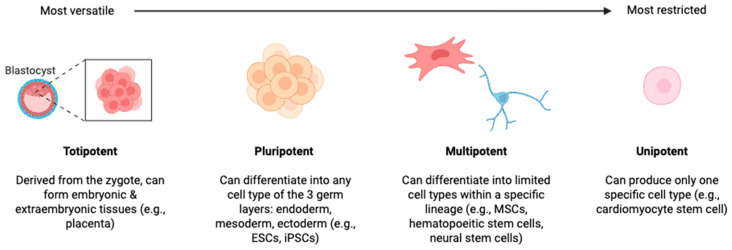
Figure depicts hierarchy of stem cells with arrow depicting versatility of differentiation. Created in BioRender. Tawfik, G. (2026) https://BioRender.com/cubcrkn, accessed on 6 March 2026.

Stem cells are commonly categorized according to their differentiation potential in a hierarchical manner (see Figure 1): totipotent, pluripotent, multipotent, and unipotent [5]. The principal human stem cell types explored for clinical and translational applications include embryonic stem cells (ESCs), induced pluripotent stem cells (iPSCs), and mesenchymal stromal/stem cells (MSCs) [7]. 

Human ESCs are derived from the inner cell mass of the blastocyst and are characterized by their pluripotency, which gives rise to derivatives of all three germ layers [8,9]. ESCs exhibit a “naïve” pluripotent state similar to that of the pre-implantation epiblast and respond robustly to differentiation cues. However, their clinical use is limited by ethical concerns and the risk of teratoma formation following transplantation [9]. 

Induced pluripotent stem cells were developed to address ethical concerns surrounding ESC use and are now considered to be one of the most promising tools in regenerative medicine [10]. iPSCs are generated through the reprogramming of somatic cells via exposure to specific transcription factors [11]. Initially developed in the early 2000s, iPSCs have since been widely applied in disease modeling, drug discovery, and therapeutic development [12]. While they abrogate ethical concerns, iPSCs still carry risks related to genetic instability and tumorigenicity [12]. This is clearly a barrier to early widespread neonatal use.

MSCs are multipotent cells derived from mesodermal tissues [10]. Common sources include bone marrow, adipose tissue, skeletal muscle, peripheral blood, umbilical cord, placenta, fetal tissue, and amniotic fluid [13]. MSCs can differentiate into adipogenic, chondrogenic, and osteogenic lineages in vitro and are commonly used due to their relative ease of isolation, expansion, and availability [14]. However, MSC populations vary widely in potency and self-renewal capacity, leading to inconsistent clinical outcomes [15]. Despite these limitations, their immunomodulatory and anti-inflammatory properties have enabled their use in treating a wide variety of disorders [16] and suggest a possible application for premature patients.

## 3. Mesenchymal Stem Cell Potential Benefit for Pre-Maturity

Premature infants face a high burden of morbidity due to the immaturity of their organs and increased susceptibility to oxidative stress, inflammation, and ischemia. Across several organ systems, sequelae of prematurity are caused by the convergence of developmental immaturity and environmental stressors during critical windows of development. Mechanisms such as interrupted morphogenesis, impaired vascular regulation, hypoxic–ischemic injury, inflammatory signaling, oxidative stress, and dysbiosis drive interrelated patterns of pathology. Stem cell-based therapies, particularly MSCs derived from umbilical cord, placenta, or bone marrow, have emerged as a promising therapeutic avenue for degenerative and inflammatory diseases that have similar underlying pathophysiology [17]. Accordingly, they may be impactful in the setting of prematurity.

### 3.1. MSC Mechanism of Benefit

Mesenchymal stromal/stem cells migrate to injured tissues in response to inflammation. MSCs were originally thought to directly replace damaged cells through their engrafting and differentiation capacity [18]. However, recent studies have revealed that MSC engraftment is transient and insufficient in duration to produce a meaningful therapeutic effect [18]. Instead, their main therapeutic effects arise from paracrine mechanisms where secreted growth factors, cytokines, chemokines, and extracellular vesicles that reduce inflammation, promote angiogenesis, and support tissue regeneration [19]. Moreover, MSCs express HLA class I but lack HLA class II, which minimizes alloreactive lymphocyte activation [20]. This immune tolerance, combined with their ability to target inflamed tissues, makes MSCs a promising therapy for inflammatory complications of prematurity.

MSC efficacy is likely the product of a transient “hit-and-run” mechanism of action [21]. In this model, MSCs briefly persist following administration, release paracrine effectors, and are rapidly cleared, while host immune and stromal cells sustain downstream reparative processes [21].

The hit-and-run concept emerged from observations that MSCs demonstrate remarkably poor engraftment following transplantation. A defining feature of intravenously administered MSC therapy is the rapid sequestration of cells within the pulmonary vasculature [22,23]. Within minutes of infusion, the majority of MSCs become trapped in lung capillaries, a phenomenon attributed to size mismatch between the cells and the pulmonary microcirculation [24]. This early localization is transient, as more than 95% of infused cells become undetectable within 72 h [25].

Human autopsy analyses similarly demonstrate minimal long-term donor cell persistence. In a landmark study by von Bahr et al., tissue samples from 18 patients who received allogeneic MSCs were examined post-mortem [26]. MSC donor DNA was detected in only 8 of 18 patients at levels ranging from 1/100 to 1/1000 cells, with detection inversely correlated with time from infusion to tissue sampling [26]. In studies tracking human umbilical cord-derived MSCs (ucMSCs) in mice, viable MSCs were detected in the lungs immediately after infusion but were nonviable by 24 h [23]. At that time, MSC remnants were detected within host monocytic populations in the lungs and liver, indicating rapid immune-mediated clearance [23]. Notably, therapeutic outcomes have not been shown to correlate with measurable engraftment, which further supports the conclusion that sustained cellular persistence is not required for clinical efficacy [26].

Despite their transient survival, MSCs secrete bioactive mediators, such as growth factors, cytokines, chemokines, extracellular matrix proteins, and extracellular vesicles that regulate angiogenesis, suppress inflammation, limit apoptosis and fibrosis, and promote tissue remodeling [22,27,28]. Even when largely confined to the lungs after systemic infusion, MSCs can exert systemic immunomodulatory effects. Single-cell RNA sequencing studies demonstrate that intravenously administered MSCs induce distinct transcriptional changes in wound macrophages at distant sites, including pro-angiogenic CD9^+^ macrophage subpopulations [29]. Secreted proteins such as *COL6A1*, *PRG4*, and *TGFB3* have been implicated in macrophage polarization and tissue repair [29].

MSCs may also exert a therapeutic effect via promotion and potentiation of native stem cell action [30]. This potential mechanism of action has been suggested in neurologic pathologies including an animal model of Alzheimer’s disease [31].

Emerging evidence further suggests that MSC apoptosis may be an integral component of the hit-and-run paradigm rather than a limitation of therapy. Transplanted MSCs rapidly activate stress and hypoxia pathways and undergo caspase-dependent apoptosis within 24–72 h [32]. In murine models of liver injury, apoptotic MSCs were detectable as early as 2 h post-injection and markedly declined by 12–24 h, with most GFP-labeled MSCs nonviable within 12 h [33]. Despite the rapid loss, both intact MSCs and apoptotic MSC derivatives attenuated tissue injury, reduced serum AST and ALT levels, suppressed proinflammatory cytokines, increased hepatocyte growth factor (HGF), and improved survival in toxin-induced acute liver injury [33]. These findings indicate that apoptosis and the associated release of immunomodulatory signals, such as phosphatidylserine may actively contribute to therapeutic efficacy [33].

Recognition of the hit-and-run mechanism carries important translational implications. If MSCs function primarily through secreted mediators and immune reprogramming, cell-free methods utilizing conditioned medium or extracellular vesicles may achieve comparable therapeutic outcomes while reducing risks associated with live cell administration [28]. Furthermore, approaches aimed at enhancing MSC paracrine potency through preconditioning, optimized timing, or route of delivery may be more impactful than efforts to improve long-term engraftment [27].

### 3.2. Extracellular Vesicles (EVs)

Extracellular vesicles (EVs) have emerged as critical mediators of the therapeutic effects attributed to MSCs, particularly in regenerative medicine and immunomodulatory therapy. EVs are nano-sized, membrane-bound particles released by virtually all cell types, including MSCs [34]. They contain complex bioactive cargo such as proteins, lipids, messenger RNA, and regulatory non-coding RNAs that reflect the physiological and functional state of their parent cells [35]. Increasing evidence supports the concept that the clinical benefits observed after MSC administration are largely driven by paracrine signaling rather than long-term engraftment or direct differentiation of transplanted cells [36]. Following systemic or local injection, only a small proportion of MSCs successfully home to injured tissues, yet significant biological effects are observed. This suggests that secreted factors, particularly extracellular vesicles, act as the principal effectors of MSC-based therapy [36]. As a result, MSC-derived extracellular vesicles are now considered potential cell-free therapeutic agents capable of reproducing many of the biological activities traditionally associated with MSC transplantation [36].

EVs derived from MSCs have several therapeutic advantages. Their small size enables a more extensive and precise distribution in vivo [37]. They have low immunogenicity, making them less likely to cause rejection reactions and they lack a cell nucleus, which decreases the risk of tumor transformation [37]. They have higher stability and can maintain their activity under different environmental conditions [38]. Furthermore, EVs are easily produced, preserved, and stored [37].

The therapeutic mechanisms of MSC-derived extracellular vesicles are largely mediated through intercellular communication and modulation of pathological signaling pathways. These vesicles can transfer functional microRNAs and regulatory proteins to recipient cells, thereby influencing gene expression programs associated with inflammation, apoptosis, proliferation, and differentiation [39]. In the context of immune regulation, MSC-EVs can suppress excessive inflammatory responses by promoting macrophage polarization toward anti-inflammatory phenotypes and reducing pro-inflammatory cytokine production [40]. Proteins such as *tumor necrosis-inducible gene 6* and specific immunomodulatory RNA cargo have been implicated in attenuating tissue injury and fibrosis [36,41]. In addition, MSC-derived extracellular vesicles contribute to tissue regeneration by enhancing angiogenesis and lymphangiogenesis through delivery of microRNAs and proteins involved in vascular signaling, including *integrin alpha-5* and *neuropilin-1*. These proteins support endothelial cell migration, tube formation, and restoration of perfusion in ischemic tissues [42]. These vesicles also support survival and proliferation of resident progenitor cells across multiple organ systems, including the liver and lung, where EV-mediated signaling has demonstrated protective effects against inflammatory damage [43].

Despite promising therapeutic potential, clinical translation of extracellular vesicle-based therapy is limited by technical and biological factors. Standardization of EV isolation, purification, and characterization methods is required to reduce product heterogeneity and ensure reproducibility across studies [44]. Large-scale manufacturing and long-term storage stability also represent major obstacles for widespread production and clinical implementation. Furthermore, optimal dosing strategies, biodistribution profiles, and pharmacokinetic behavior of EVs in vivo are not yet established. Ongoing research is exploring bioengineering approaches to enhance EV targeting efficiency and therapeutic cargo loading, including manipulation of microRNA content within MSC-derived vesicles as a strategy to improve regenerative outcomes [39]. As our understanding of EV biology evolves, extracellular vesicles derived from MSCs are expected to play an increasingly important role as a safer and more controllable alternative to conventional cell-based therapies.

## 4. Clinical Applications

The ultimate goal of stem cell therapy is to heal damaged tissue that cannot repair itself [6]. Stem cells have shown promise across multiple tissue types, including mesoderm-derived tissues (bone, cartilage, muscle), nervous tissue, skin, and organs such as the liver, gastrointestinal tract, vasculature, and lungs [10]. This literature suggests possible therapeutic applications for the complex pathologies observed in prematurity. Successful clinical applications require a clear understanding of cell derivation, cell processing, timing of administration, route of administration, and patient selection.

### 4.1. Clinical Applications—Cell Selection and Administration

When using iPSCs or MSCs, there are two possible sources of cells: autologous or allogenic. Autologous cell sources refer to stem cells obtained from the same individual who will later receive them. Therefore, autologous cells are inherently immunocompatible, which minimizes the risks of immune rejection, graft-versus-host disease (GVHD), and the need for immunosuppressive therapy [45]. Autologous therapies also raise fewer ethical and regulatory concerns. However, cell quality and quantity are highly dependent on patient age, health, and disease status, and preparation can be time-consuming due to the need for cell isolation and expansion.

Allogeneic stem cells are derived from genetically distinct donors, such as unrelated individuals or family members. These therapies offer the advantage of standardized, high-quality cell lines and “off-the-shelf” availability, which is valuable for acute conditions requiring rapid intervention [45]. However, allogeneic transplantation carries increased risks of immune rejection, alloimmunization, and GVHD, and oftentimes necessitates immunosuppressive therapy [46]. MSCs are notable for their relatively low immunogenicity, and allogeneic MSC therapies have encouraging safety profiles in clinical studies [16]. Despite extensive research, the advantages and risks of autologous versus allogeneic MSC therapies continue to be debated, particularly with regard to donor heterogeneity and long-term immune responses [16].

The route of administration strongly influences biodistribution, engraftment, and mechanism of action. Intravenous delivery is minimally invasive but subject to pulmonary first-pass effects, whereas intra-arterial and local injections improve targeting at the cost of increased procedural risk. Intrathecal administration is commonly used for neurological indications.

The timing of stem cell delivery is a critical determinant of therapeutic efficacy. In acute injury settings—such as myocardial infarction or ischemic stroke—early intervention may improve outcomes by modulating inflammatory cascades and preventing irreversible tissue loss [47]. However, overly rapid administration can also expose transplanted cells to hostile microenvironments characterized by oxidative stress or inflammation, reducing survival [48]. In chronic degenerative diseases (e.g., osteoarthritis, heart failure), treatment timing is often guided by the stage of disease progression, with mid-stage pathology generally considered optimal because tissue architecture may still support engraftment and functional integration [49].

Furthermore, there are instances in which stem cell use may actually be detrimental. A study analyzing the results of 904 patients undergoing stem cell transplantation for follicular lymphoma found that both autologous and allogeneic transplantation induced long-term disease control in patients with a major benefit when performed early in the course of disease but led to a dismal course when given for refractory disease [50]. Overall, the ideal timing depends on balancing the disease and its biochemical signals with the transplanted cells’ ability to survive and differentiate. Determining optimal dosing remains challenging due to variability in cell type, viability, and functional potency [51]. While higher doses may enhance therapeutic effects, excessive dosing can increase immune reactions, microvascular obstruction, or uncontrolled proliferation [52].

### 4.2. Clinical Applications—Patient Selection

Patient-related factors are central to selection for stem cell therapies. Autologous stem cell transplantation (ASCT) is an established treatment for malignant (e.g., multiple myeloma [53]) and non-malignant (e.g., sickle cell disease, thalassemia [54]) disorders in both adult and pediatric populations [55,56,57]. ASCT provides deep, durable remissions and, in many settings, standard-of-care consolidation following induction therapy. Selection of candidates for ASCT requires a careful balance between anticipated therapeutic benefit and the risks associated with high-dose conditioning and transplant-related toxicity [55].

Adequate organ function (cardiac, pulmonary, hepatic, and renal) is essential to tolerate high-dose chemotherapy and prevent severe complications, and significant comorbidities or uncontrolled infections generally contraindicate ASCT [55]. Recent practice has shifted away from strict age cut-offs to assessment tools such as performance status and frailty assessments. These tools help determine candidacy since older adults can also safely undergo ASCT if selected appropriately [55]. Patient preference and psychosocial support are also key, as transplant therapy demands robust support systems for patients [58].

In summary, ASCT candidate selection is individualized; it requires integrating disease responsiveness, overall health and organ function with shared decision-making to optimize outcomes while minimizing risks [54]. Conducting clinical trials in children can help researchers to discover the best way to treat pediatric diseases, thus dramatically improving their health care [59]. However, there are several scientific, ethical, and practical difficulties that make conducting clinical trials in children challenging. Higher quality and larger quantity clinical trials in children are needed to expand our understanding of the treatment of pediatric diseases using ASCT [59].

## 5. Stem Cell Therapies for Prematurity

There is a growing body of both pre-clinical and clinical evidence supporting clinical utility for the application of MSCs for the treatment of prematurity-associated pathology. An overview of proposed MSC mechanisms of action and clinical outcomes for various sequelae of prematurity is summarized in Table 1. An overview of applications of MSC therapies in the context of preterm neurologic insults is presented below.

### 5.1. Central Nervous System Pathology

MSCs may modulate neurological pathologies in the setting of preterm birth. Neurologic vulnerability is highest in very preterm (<32 weeks) and extremely preterm (<28 weeks) infants. Delivery during this time frame exposes the immature periventricular white matter vasculature to hypoxic–ischemic injury. Pre-oligodendrocytes (pre-OLs) ensheath developing axons between 24 and 40 weeks of gestation; this process does not finish until after term [60]. Ischemic, free-radical, and glutamate-mediated cell death have been shown to be primarily mediated by inflammatory microglia, leading to pre-OL cell death [61]. In premature infants, the resulting diffuse white matter injury (dWMI) and widespread hypomyelination are collectively referred to as encephalopathy of prematurity (EoP), which often presents as broad neurodevelopmental deficits.

### 5.2. Pre-Clinical Evidence

There is pre-clinical evidence supporting a role for MSC therapies for neurodevelopmental pathology. By promoting OL survival and reducing inflammation, MSCs facilitate neuro-regeneration and myelination [62]. In a rat model of subcortical stroke, intravenous MSCs were shown to increase OL proliferation in lesion areas, higher myelin production, and smaller functional deficit [63]. Intranasal MSC delivery after 14-month follow up of hypoxic–ischemic injury and in models of dWMI has shown improved motor behavior, differentiation of neural stem cells and rescue of OL maturation, and decreased inflammation [62,64].

Preclinical studies have shown that hypothermia combined with umbilical cord blood-derived mesenchymal stromal cells administration significantly reduce cerebral infarct volume and improves motor outcomes in rat models of hypoxic–ischemic encephalopathy (HIE) [17]. These benefits are mediated by MSC-derived paracrine signaling, including the release of *IGF-1*, *basic fibroblast growth factor*, neural cell adhesion molecules, nerve growth factors, and anti-inflammatory cytokines, which all promote neurogenesis and tissue repair [17].

### 5.3. Clinical Evidence

Several clinical trials published within the last 10 years have reported the use of cell transplants for neonatal encephalopathy (NCT00593242 United States, NCT02256618 Japan, NCT03635450 United States, NCT04261335 Japan) [65].

One study examined neonates born at least 35 weeks of gestation with moderate or severe HIE with or without treatment with autologous umbilical cord blood (UCB) cells [66]. A total of 105 infants were examined: 23 infants were treated with UCB infusions, and 82 infants met the same HIE criteria but did not have UCB available. Concurrent therapeutic hypothermia and dosages of stem cells contained 1.5 × 10^7^ cells/kg, and up to 4 doses (at birth, 24, 48, and 72 postnatal hours) were given. This study was completed before FDA regulations prompted a maximum of 2 infusions within 48 postnatal hours. This study demonstrated that UCB collection, processing, and infusion were feasible and safe. Preliminary data suggested potentially improved neurodevelopmental outcomes after one year of age [66].

In a similar feasibility and safety study, researchers enrolled 6 infants born by cesarean section ≥36 weeks gestation with moderate to severe encephalopathy. The single arm intervention consisted of three IV autologous umbilical cord blood cell infusions at 12–24, 36–48, and 60–72 h after birth. All participants also received therapeutic hypothermia. Researchers demonstrated that adequate UCB volume collection was feasible. They found that all infants survived to 30 days without circulatory or respiratory support, and four of six infants showed normal neurological development at 18 months [67].

**Figure 2:**
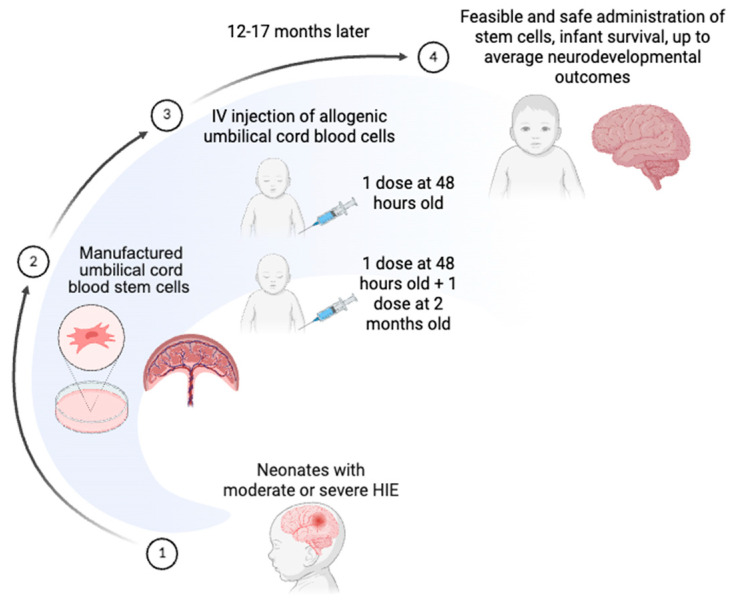
Figure depicts timing of administration of allogenic hCT-MSC therapy for neonatal HIE. Created in BioRender. Tawfik, G. (2026) https://BioRender.com/cubcrkn, accessed on 6 March 2026.

IV infusions of allogeneic human umbilical cord tissue-derived mesenchymal stromal cells (hCT-MSC) were studied in a prospective pilot, open-label, phase I clinical trial involving six neonates with moderate or severe HIE. Infants were eligible if they were at least 35 weeks of gestation and therapeutically cooled for the first 72 h after birth. hCT-MSCs were manufactured from umbilical cord tissue that was donated to the Carolinas Cord Blood Bank at Duke University Medical Center. Dosages of hCT-MSC consisted of 2 million cells/kg administered IV over 30 min. Six infants received a single dose during therapeutic hypothermia within 48 h, and two received an additional dose at 2 months of age. At follow up (12–17 months), all infants tested for within the average to low-average ranges on the Bayley-III Toddler Development Scales (see Figure 2). From this study, Cotten et al. concluded that the IV administration of allogenic hCT-MSCs in neonates with moderate to severe HIE is feasible and safe, and that using an off-the-shelf cellular product allows for improved access to infusion products [68]. Importantly, one major safety issue was the development of anti-class I HLA antibodies in 5 of 6 infants, which could limit donors for future transplantation.

Therefore, MSC-based therapies may have a role in treating encephalopathy of prematurity. Early-phase clinical studies suggest that administration of autologous and allogeneic cell products is feasible and safe during the neonatal period. Data from existing trials, however, are limited by small sample size, heterogeneous study designs, and short time frames for patient follow up.

### 5.4. Technical Barriers

The clinical implementation of MSC therapies in premature neonates is limited further by manufacturing, quality control, and economic barriers. Clinical translation of stem cell therapies requires standardized in vitro characterization and good manufacturing practices (GMP) compliant processes [69,70]. For example, scaling MSCs production to clinically relevant doses is challenging, and traditional two-dimensional culture methods are labor-intensive [70]. Large-scale bioreactors are more efficient; however, they are more costly and require specialized skills [70]. Cryopreservation of MSCs is essential for large-scale storage and production, but it can negatively affect cell viability, phenotype, and therapeutic efficacy [70]. Furthermore, quality control issues include cellular variability and instability, contamination risks, the absence of suitable terminal sterilization methods, and the lack of standardized processing protocols [71].

Economic barriers remain a major limitation to MSC clinical translation, as manufacturing costs alone range from approximately $10,000 to $30,000 per patient dose or $15,000–$30,000 per 1–5 million GMP MSCs/kg, with total costs increasing further when clinical delivery, follow-up, or genetic modification are included [70,72]. Without established reimbursement strategies and a clear demonstration of cost-effectiveness, access will be limited to affluent patients and higher-income countries, which exacerbates current global health disparities [70,72].

Finally, there are several ethical barriers for MSC therapy in neonates. Informed consent is a major ethical concern and barrier to translation. Oftentimes, parents must make decisions about experimental interventions under significant emotional stress, necessitating voluntary and fully informed consent processes. Unresolved ethical issues for MSC utilization include donor rights, tissue ownership, and biobanking.

## 6. Future Directions

The future of mesenchymal stem cell therapy lies in overcoming current limitations through technological innovation and standardized clinical practices. Manufacturing is shifting from autologous to allogeneic off-the-shelf products to improve scalability and reduce costs, while genetic engineering techniques, including CRISPR modification, are being explored to enhance MSC therapeutic properties [73]. Cell-free alternatives, particularly MSC-derived extracellular vesicles and exosomes, are emerging as promising therapies that retain immunomodulatory effects while avoiding concerns related to cell viability and engraftment [74].

Over 1200 clinical trials are currently investigating MSC therapy [75]. The main challenges limiting clinical translation are MSC heterogeneity, immunocompatibility, and long-term preservation. Standardization of clinical protocols, including dosing regimens, timing of administration, and delivery routes, is essential to improve reproducibility across trials [76]. Equally important is selecting the appropriate MSC product for each disease indication, with consideration of the optimal administration route and mechanism of action [77].

Despite these challenges, there is growing evidence for benefit and the safety profile of MSC therapy. The risk of teratoma formation and ectopic tissue complications with MSC therapy appears minimal based on current clinical evidence [78]. A comprehensive meta-analysis of 62 randomized clinical trials involving 3546 participants found no serious adverse events such as malignancy associated with MSC administration, demonstrating consistent safety across diverse disease populations [79]. A large retrospective study of 2504 patients receiving intravenous adipose-derived MSC therapy reported a major adverse event rate of only 0.2%, with no severe adverse events observed during follow-up [80].

## 7. Conclusions

Despite these potential barriers to translation, the benefits of MSC therapies have been demonstrated across diseases associated with preterm birth and studies of mesenchymal stromal/stem cells have become recognized as a developing area of therapeutic investigation. MSCs exert not only regenerative effects but also anti-inflammatory and reparative mechanisms that limit immune-mediated injury while promoting angiogenesis, tissue maturation, and facilitate repair. Overall, this suggests clear potential for benefit in the context of prematurity.

However, substantial barriers to wide-spread application must be overcome. The pace of technical advancement, the continued collection of data including clinical studies and the substantial burden of prematurity suggest a likely growing role for MSC therapies in clinical practice.

## Figures and Tables

**Table 1:** Organ-specific effects of MSCs in diseases of prematurity.

Associated Disease	Actions	Outcomes
Encephalopathy of prematurity	Secretion of neurotrophic factors (e.g., IGF-1, bFGF). Anti-inflammatory signaling. Support of angiogenesis and myelination.	Reduced cerebral infarct volume. Enhanced neurogenesis and tissue repair. Improved motor outcomes.
Intraventricular hemorrhage	Release of angiogenic factors (VEGF, bFGF, angiopoietin-1). Suppression of microglial activation and pro-inflammatory cytokines. Reduction in oxidative stress and apoptosis.	Reduced progression of hemorrhage. Reduced ventricular dilation. Improved neurocognitive outcomes. Promoted angiogenesis, myelination, neuronal rewiring, tissue repair. Reduced gliosis and cell death. Supported neuronal survival.
Bronchopulmonary dysplasia	Suppression of inflammatory mediators (IL-6, TNF-α, TGF-β). Secretion of pro-angiogenic and alveolar-supportive factors (VEGF, HGF, KGF). Inhibit fibroblast activation and collagen deposition.	Reduced neutrophilic infiltration attenuating ventilator- and oxygen-induced lung injury. Improved alveolarization and tissue repair. Reduced fibrosis and inflammation. Reduced disease severity.
Necrotizing enterocolitis	Attenuation of TLR4-mediated inflammation. Promotion of reparative macrophage polarization. Secretion of epithelial growth and anti-inflammatory factors (EGF, HGF, IL-10).	Suppressed mucosal infarction and necrosis. Enhanced epithelial regeneration and barrier integrity. Accelerated mucosal healing. Improved intestinal perfusion. Reduced oxidative stress and apoptosis.

## Data Availability

No new data were created or analyzed in this study. Data sharing is not applicable to this article.

## Abbreviations

The following abbreviations are used in this manuscript:

MSC	Mesenchymal stem cell
BPD	Bronchopulmonary dysplasia
NEC	Necrotizing enterocolitis
ESCs	Embryonic stem cells
iPSCs	Induced pluripotent stem cells
HLA	Human leukocyte antigens
DNA	Deoxyribonucleic acid
ucMSCs	Umbilical cord-derived mesenchymal stromal cells
RNA	Ribonucleic acid
HGF	Hepatocyte growth factor
EVs	Extracellular vesicles
IGF	Insulin-like growth factor
bFGF	Basic fibroblast growth factor
VEGF	Vascular endothelial growth factor
IL	Interleukin
TNF	Tumor necrosis factor
TGF	Transforming growth factor
KGF	Keratinocyte growth factor
TLR	Toll-like receptor
EGF	Epidermal growth factor
GVHD	Graft-versus-host disease
OLs	Oligodendrocytes
ASCT	Autologous stem cell transplantation
dWMI	Diffuse white matter injury
EoP	Encephalopathy of prematurity
HIE	Hypoxic–ischemic encephalopathy
UCB	Umbilical cord blood
FDA	Food and Drug Administration
hCT-MSC	Human umbilical cord tissue-derived mesenchymal stromal cells
GMP	Good manufacturing practices
CRISPR	Clustered Regularly Interspaced Short Palindromic Repeats
